# Case Report: Laryngotracheal Post-Intubation/Tracheostomy Stenosis in COVID-19 Patients

**DOI:** 10.3389/fsurg.2022.874077

**Published:** 2022-04-25

**Authors:** Ilaria Onorati, Nicolas Bonnet, Dana Mihaela Radu, Olivia Freynet, Patrice Guiraudet, Marianne Kambouchner, Yurdagul Uzunhan, Elie Zogheib, Emmanuel Martinod

**Affiliations:** ^1^Department of Thoracic and Vascular Surgery, Assistance Publique-Hôpitaux de Paris, UFR de Santé Médecine Biologie Humaine, Université Sorbonne Paris Nord, Bobigny, France; ^2^Department of Intensive Care Medicine, Assistance Publique Hôpitaux de Paris, UFR de Santé Médecine Biologie Humaine, Université Sorbonne Paris Nord, Bobigny, France; ^3^Department of Pulmonology, Assistance Publique Hôpitaux de Paris, UFR de Santé Médecine Biologie Humaine, Université Sorbonne Paris Nord, Bobigny, France; ^4^Department of Pathology, Assistance Publique-Hôpitaux de Paris, Bobigny, France; ^5^Department of Anesthesiology, Assistance Publique Hôpitaux de Paris, UFR de Santé Médecine Biologie Humaine, Université Sorbonne Paris Nord, Bobigny, France

**Keywords:** laryngotracheal stenosis, COVID-19, post-intubation stenosis, post-tracheostomy tracheal stenosis, prone position (PP)

## Abstract

**Introduction:**

The novel Coronavirus disease 2019 (COVID-19), which is caused by severe acute respiratory syndrome coronavirus 2 (SARSCoV-2), has spread rapidly to become a major global public health emergency since March 2020. Laryngotracheal stenosis (LTS) has been observed more frequently since the onset of the COVID-19 pandemic.

**Methods:**

All patients referred to our 24/7 Airway Diseases Center for laryngotracheal post-intubation/tracheostomy stenosis from May 2020 to May 2021were evaluated retrospectively. Patient data on comorbidities, diagnosis, type of procedures, lengths of ICU stay and invasive mechanical ventilation, medical treatment, and the severity of illness were recorded.

**Results:**

This case series included nine patients (five women and four men), with a mean age of 52.9 years, most with a BMI >30, all with a severe illness revealed by the Simplified Acute Physiology Score (SAPS) II >31. From May 2020 to May 2021, 21 procedures were performed on seven patients, consisting of bronchoscopic rigid interventions, T-tube Montgomery tracheostomy, and one cricotracheal resection with end-to-end anastomosis. Histologic examination of tracheal biopsies showed an inflammatory state of the airway mucosa. Two patients only had medical therapy.

**Discussion and Conclusions:**

Pneumonia caused by SARSCoV-2 can lead to severe acute respiratory distress syndrome (ARDS) requiring invasive mechanical ventilation. The time of intubation, the drugs used, the prone position, comorbidities (diabetes, obesity), and the inflammatory state of the upper airways linked to the viral infection, predispose to an increased tendency to stenosis and its recurrence. A conservative approach with medical and endoscopic treatment should be preferred in case of persistence of local airways inflammation. Further studies with a larger sample of patients will help to a better understanding of the disease, reduce the prevalence, and improve its treatment.

## Introduction

The novel Coronavirus disease 2019 (COVID-19), which is caused by severe acute respiratory syndrome coronavirus 2 (SARSCoV-2), has spread rapidly to become a major global public health emergency since March 2020 (1). In a minority of patients, COVID-19 results in severe illness compounded by serious respiratory complications, sepsis, septic shock, multiple organ failure, and even death (2). Eighty percent of COVID-19 patients admitted to intensive care units (ICU) require prolonged mechanical ventilation, which implies the possibility of undergoing a tracheostomy for weaning from ventilatory assistance.

Probably due to the high rate of invasive mechanical ventilation (IVM) and the inflammatory state of the upper airways linked to the viral infection, laryngotracheal stenosis (LTS) has been observed since the onset of the COVID-19 pandemic. Since May 2020, nine patients were referred to our center for symptomatic LTS.

## Methods

The present study consisted of a retrospective review of all patients referred to our 24/7 Airway Diseases Center for malignant or benign lesions, Department of Thoracic Surgery, Avicenne Hospital, in the period from May 2020 to May 2021, affected by laryngotracheal post-intubation/tracheostomy stenosis after documented SARS-CoV-2 infection with critical illness requiring invasive mechanical ventilation.

All patients with suspected or documented post-intubation/tracheostomy tracheal stenosis were evaluated with CT of the neck and chest and flexible fiberoptic bronchoscopy.

Patient data on comorbidities, diagnosis, type of procedures, lengths of ICU stay and IMV, medical treatment, the severity of illness were recorded.

Rigid bronchoscopic procedures were realized according to our protocol: patients were pre-oxygenated by High flow nasal-canula oxygen therapy (HFNO) at 100% fractional inspired oxygen (FiO_2_) administration; after general anesthesia by target-controlled infusion of propofol/remifentanil, patients were intubated (rocuronium) and conventional ventilation was performed (5–7 ml/Kg tidal volume, respiratory rate 10–12/min and FiO_2_^<60%^ prior to bronchoscopy.

The endoscopic procedures performed depend on the type of laryngotracheal lesion and they consisted of tracheal dilatation by tracheobronchial balloon (Pulmonary Boston®), resection of granulomas, thermocoagulation, placement of the tracheal stent, and biopsy of tracheal abnormal tissue for histological examination.

The medical therapy consisted of corticosteroid treatment (systemic and/or inhaled aerosol) and antibiotic therapy.

This study was approved by the Ethics Committee of Avicenne Hospital, “CLEA”, on November 15, 2021 (ID number CLEA-2021-224). The patient's consent for the study was also obtained.

## Results

From May 2020 to May 2021, 104 rigid bronchoscopies were performed in our 24/7 Airway Diseases Center. Twenty procedures (20.8%) were done for laryngotracheal post-intubation and/or tracheostomy stenosis after COVID-19 pneumonia, on seven patients.

There were five women and four men with a mean age of 52.9 years (ranging from 24 to 72 years). Patients' characteristics including medical history, previous treatment, and ICU staying, are summarized in Table 1. Obesity, with a BMI >31 was found in most of them. All of them had been hospitalized in a critical care unit in the period of severe pulmonary infection, with a mean length of stay of 39.3 days (ranging from 13 to 95 days). All patients were intubated, two of them required re-intubation because of laryngeal edema and one patient for extensive tracheobronchomalacia. All patients presented a severe illness with a Simplifies Acute Physiologic Score (SAPS) II > 31. Seven of the nine tracheostomies were elective procedures, realized during the ICU hospitalization to optimize weaning from the ventilatory support. In one case, the patient needed a tracheostomy because bilateral laryngeal nerve paralysis occurred 1 month after discharge from ICU. The time between the end of mechanical ventilation and the onset of respiratory symptoms due to stenosis was short in most of the cases: less of 1 month for seven patients and 6 months for patients 5 and 6, and consisting of progressive dyspnea, weaning, or tracheostomy cannula ablation failure.

**Table 1:** Patients' characteristics.

**Sex, age (years)**	**Medical history**	**BMI**	**Previous treatment**	**NIV before IMV**	**Length of IMV**	**Prone position**	**Adjuvant drugs**	**Corticosteroids during ICU stay**	**ICU length of stay**	**SAPS II score**
1 M, 24	None	48	None	None	12	Yes	azithromycin	Yes	13	34
2 M, 53	Diabetes	24	OAD and insulin	None	21	Yes	hydroxychloroquine	No	62	44
3 F, 72	Asthma	33	Bronchodilators	HFNO	12	Yes	Hydroxychloroquine azithromycin	Yes	22	35
4 F, 40	Tracheal stenosis (Wegener granulomatosis), HIV, asthma	28	Anti-cd20 monoclonal antibodies (Rituximab) and corticosteroids	HFNO	11	No	None	Yes	13	31
5 M, 58	Hypertension, Diabetes	40	OAD	None	15	No	None	No	21	-
6 M, 57	Kidney failure, Arterial hypertension, Diabetes	31	Insulin	NIV	15	Yes	Chloroquine azithromycin	No	18	48
7 F, 60	Multiple sclerosis, hypothyroidism	35	Levothyrox	None	47	No	None	Yes	50	33
8 M, 51	None	34	None	HFNO-VV ECMO	86	Yes	None	Yes	95	55
9 F, 61	Arthrosis	31	None	NIV/HFNO	43	No	Tocilizumab	Yes	60	-

*BMI, body mass index; NIV, Non-invasive ventilation; IMV, invasive mechanical ventilation; ICU, Intensive care unit; SAPS II, Simplifies Acute Physiologic Score; OAD, Oral anti-diabetic drugs; HFNO, High flow-nasal canula oxygen therapy; HIV, Human immunodeficiency virus infection; VV–ECMO, VenoVenous Extracorporeal Membrane Oxygenation*.

The mean time between intubation and elective tracheostomy was 24.75 days (11–45 days).

Characteristics related to LTS causes, tracheostomy, bronchoscopic procedures, and outcomes are described in Table 2.

**Table 2:** Details of LTS, treatment and patients' outcomes.

	**Cause of LTS**	**Type of LTS**	**Tracheostomy**	**Other COVID-19 complications**	**N**°**BP**	**Type of intervention**	**Outcomes (Follow-up at February 2022)**
1	Post-intubation	Laryngotracheal stenosis post intubation	Surgical tracheostomy after several bronchoscopic dilatations	None	7	Tracheal dilatations Tracheotomy	Alive with Montgomery T-tube
2	Post-tracheostomy	Tracheal stenosis post tracheostomy	Percutaneous tracheostomy over prolonged intubation	Right lung empyema requiring surgical decortication	5	Tracheal dilatations Tracheotomy	Alive with Montgomery T-tube
3	Post-tracheostomy	Subglottic stenosis post tracheostomy	Surgical tracheostomy over prolonged intubation	Myocarditis	4	Tracheal dilatations Tracheal stent (silicone)	Alive without tracheostomy
4	Post-intubation	Recurrence of spontaneous laryngeal stenosis	Percutaneous tracheostomy over prolonged intubation	None	0	NA	Alive without tracheostomy
5	Post-tracheostomy	Tracheal stenosis post tracheostomy	Surgical tracheostomy for laryngeal edema requiring reintubation	None	0	NA	Alive without tracheostomy
6	Post-tracheostomy	Tracheal stenosis post tracheostomy	Surgical tracheostomy for bilateral paralysis of vocal cords	Bilateral paralysis of vocal cords	1	Tracheal dilatation and granuloma's resection	Alive without tracheostomy
7	Post-intubation	Subglottic stenosis with extensive tracheobronchomalacia	Surgical tracheostomy	None	2	Tracheal stenting for tracheomalacia Dilatation of subglottic stenosis	Alive without tracheostomy
8	Post-tracheostomy	Tracheal stenosis post tracheostomy (Granuloma)	Percutaneous tracheostomy over prolonged intubation	None	1	Tracheal dilatation and granuloma's resection Cricotracheal resection and end-to-end anastomosis	Alive without tracheostomy
9	Post-tracheostomy	Tracheal stenosis post tracheostomy (Granuloma)	Percutaneous tracheostomy over prolonged intubation	None	1	Granuloma's resection	Alive without tracheostomy

*LTS, laryngotracheal stenosis; BP, bronchoscopic procedures*.

**Figure 1:**
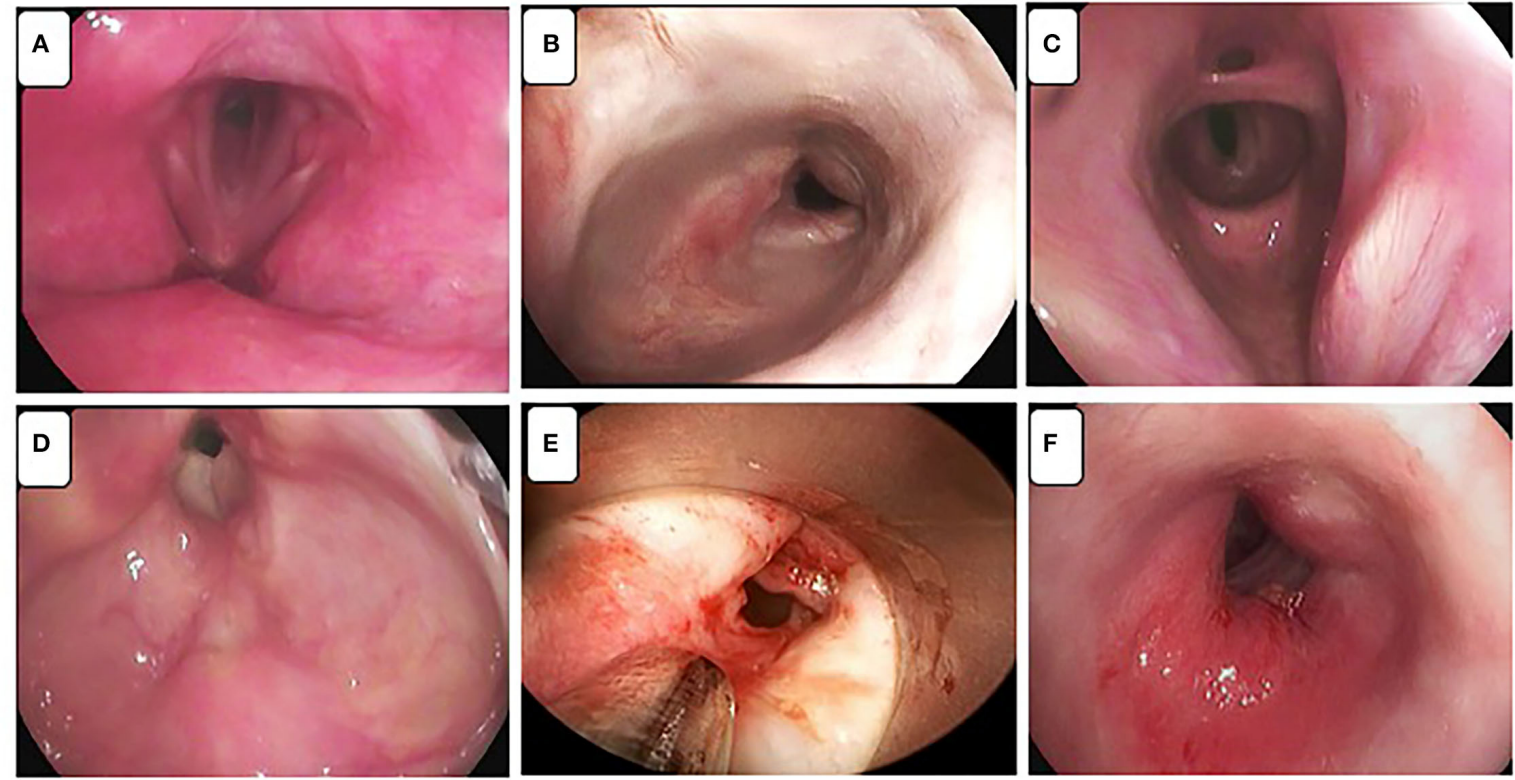
Different patterns of laryngotracheal stenosis (LTS) associated with endotracheal intubation or tracheostomy placement. **(A)** Patient 1: cicatricial subglottic stenosis due to long-term intubation. **(B)** Patient 2: tracheal stenosis resulting from tracheostomy cuff injury. **(C)** Patient 3: same condition at different levels of the airway with a complex diaphragm stenosis. **(D)** Patient 4: inflammatory subglottic stenosis with granulomatous' tissue. **(E)** Patient 6: subglottic stenosis after dilatations. **(F)** Patient 8: subglottic stenosis with oedema and inflammatory aspect of vocal cords.

The endoscopic aspect of some of the LTS is shown in Figure 1.

Severe acute respiratory syndrome coronavirus 2 reverse transcription-PCR (RT-PCR) tests were performed routinely the day before the procedure for all patients and all were negative.

During the invasive procedures, all personnel in the operating room were equipped with an FFP2 face mask, disposable gloves, and a cap. Furthermore, the bronchoscopist and bronchoscopy assistants were dressed in a surgical gown and eye protection (reusable safety glasses or face shield). The symptoms of the health care workers who performed the procedures were monitored closely. No infection occurred in the weeks following the procedure.

Follow-up of patients included a fiberoptic flexible bronchoscopy 1 month after the procedure or earlier in case of recurrence of respiratory symptoms.

Patients 1, 2, and 3 required repeated interventions because of recurrence of symptomatic tracheal stenosis. Two of them needed a Montgomery T-tube to treat the subglottic stenosis. In one case (patient 1), the T-tube was removed 1 month after its placement because of repeated occlusion of the endotracheal lumen and replaced by a standard tracheostomy tube. The same patient, despite long-term treatment by corticosteroids and targeted antibiotic therapy, still suffers from important tracheal inflammation and recurrent stenosis. This patient, the youngest of the series, is awaiting endoscopic treatment for obesity and undergoing nutritional rehabilitation to better prepare for further airway surgery. Recently, in February 2022, he required a new session of endoscopic dilatation by rigid bronchoscopy and placement of Montgomery T-tube to treat the subglottic stenosis.

**Figure 2:**
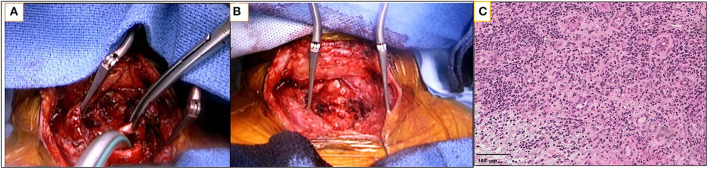
Surgical view of cricotracheal resection and end-to-end anastomosis and histological examination of a tracheal biopsy. **(A)** Transverse cervical incision: after tracheotomy, distal trachea is intubated from the surgical field with a Montandon cannula, and the oral tracheal tube is retracted. **(B)** Final aspect of end-to-end cricotracheal anastomosis. **(C)** Microscopic image (Hematoxylin-eosin staining 20 × magnification) of tracheal biopsy of patient 3. The tissue is surrounded by lymphocytes and plasma cells, indicating active inflammation.

Only one patient (patient 8) in our series could benefit from surgical resection of the lesion. He underwent cricotracheal resection and end-to-end-anastomosis (Figures 2A,B), 2 weeks after the end of corticosteroid therapy and 1 month after the endoscopic procedure for tracheal dilatation and granulomatous tissue resection.

All histological examinations of tracheal biopsies showed altered inflammatory neutrophils, a granulation tissue surrounded by lymphocytes and plasma cells, indicating active inflammation (Figure 2C). The histologic aspect of tracheal resection showed localized tracheal stenosis related to the presence of small “chronic” non-fistulized abscesses, responsible for osteochondritis with self-sustaining phenomena of bone and cartilage lysis.

## Discussion

Our series is a heterogeneous group of patients with COVID-19 with postintubation/tracheostomy LTS. In our department, we have at our disposal a full arsenal and experience in the treatment of tracheobronchial diseases ranging from endoscopic procedures to interventional bronchoscopy (3), to resection-anastomosis up to airways replacement by stented aortic matrices (4). By far, we adopted a conservative attitude in most of the cases of COVID-19 LTS. In our opinion, this was the best solution (even though sometimes temporary) for these lesions.

In literature, post-intubation tracheal stenosis is the most common type of tracheal stenosis (5). With the introduction of an endotracheal tube with a high-volume low-pressure cuff in the 1970s, the risk of post-intubation tracheal stenosis has decreased, but its incidence is still reported to range from 6 to 21% (6, 7), but only 1–2% of patients present with severe or symptomatic clinical presentations. The standard care for tracheal stenosis is tracheal resection with end-to-end anastomosis; however, not all patients seem to benefit from the surgery (6, 8, 9). Endoscopic procedures and medical treatment (corticosteroids and antibiotic therapy) can be used first, before open surgery (10, 11), but patients may require repeated interventions. If stenosis recurs after a primary intervention, treatment becomes complicated, and the prognosis tends to be poor (12).

Pneumonia caused by SARSCoV-2 (COVID-19) can lead to severe Acute Respiratory Distress Syndrome (ARDS) requiring invasive mechanical ventilation in 80% of patients admitted at the ICU (2). COVID-19-related ARDS is associated with the severe impairment of lung ventilation/ perfusion matching, resulting from a defect of hypoxic pulmonary vasoconstriction and presence of thrombi in the pulmonary microcirculation, leading to high intra-pulmonary shunt and dead space, respectively (13–15). In the literature, we find some case reports (16, 17) regarding tracheal stenosis in patients with COVID-19, suggesting a possible role of infection in the genesis of disease. In a recent paper published by the Laryngotracheal stenosis Committee of the European Laryngological Society, the authors alerted the medical community about the possible rise in the number of COVID-19-related LTS to prevent this complication and to refer the patients to specialized airway centers (18).

Peris et al. studied the pathological findings in organs and tissues from biopsies and autopsies of severe COVID-19 cases and for the respiratory tract, inflammation was found in 100% of all the specimens analyzed (19). They described pharyngeal hyperemia, tracheitis, and bronchial mucosa edema. A persistence of this inflammatory state during and after intubation and/or tracheostomy could explain the higher predisposition to stenosis.

In our series, we found a higher number of tracheal post-intubation and tracheostomy stenosis during the first wave of the pandemic (six out of nine patients). It is possible that the changing in medical treatment and the increased use of non-invasive ventilation (NIV) after the first period of SARS-Cov-2, could partially explain a lower incidence of LTS in these patients during the second and third wave of the pandemic. Indeed, the management of COVID-19 has substantially changed over time. During the first period of the pandemic, the trend was to adopt an early intubation strategy, because of the hypothetic risk of transmission to healthcare workers, restricting the use of HFNO and limiting the flow rate to 30 L/min for critically ill patients with COVID-19 (20, 21). Regarding medical treatment, antibiotic and/or antiviral therapies were systematically used. The trends in hydroxychloroquine use showed a rapid increase during February and March 2020, followed by a similarly rapid decline in May that continued until the end of the year (22). Corticosteroids have been little used in the first few months of the pandemic and, after the Recovery trial report in June 2020 (23) that showed a reduction in mortality associated with dexamethasone, its administration rapidly increased (22). Corticosteroids are now part of the standard treatment of oxygen-dependent patients with COVID-19 and may reduce the incidence of LST. Due to the high mortality rate in patients needing invasive mechanical ventilation, and after the scientific evidence that generation and dispersion of bio-aerosols *via* HFNO showed a similar risk to standard oxygen masks, HFNO was accepted as current practice for hypoxemic COVID-19 patients to avoid intubation (20, 24, 25). That could also contribute to a reduction in the rate of LST.

Early prone position (PP) has been commonly used in critically ill patients affected by the COVID-19, both in patients receiving invasive mechanical ventilation (13, 26), and in those spontaneously breathing (27). Already used for non-COVID-19 severe ARDS, PP can improve oxygenation and survival as compared to a supine position (28) thanks to the recruitment of atelectatic dorsal lung areas and the redistribution of lung ventilation toward still well-perfused areas, but repeated microtraumas could play a possible role in the pathogenesis of the stenosis.

About the treatment of LTS in COVID-19 patients, two questions arise: when and which treatment to choose? In our referral center for airway disease, we chose a conservative attitude in the first place, mainly due to the aspect of LTS. In all patients, we found an extremely inflammatory aspect of the upper airways' mucosa and therefore chose to proceed to endoscopic treatment with rigid bronchoscopy in 7 patients. In two cases, endoscopic treatment was used as a possible bridge to surgical treatment. In patient 1, open surgery was considered several times but not carried out due to the persistent inflammation state, despite optimal medical treatment. We could perform surgery only for one patient of the series (patient 8).

In our series, due to the high prevalence of tracheostomy, it is difficult to know whether LTS is subsequent to intubation or the tracheostomy itself. In post-tracheostomy stenosis, the type of tracheostomy, surgical or percutaneous (3 vs. 3), appears to be unrelated to an increased tendency to stenosis.

Risk factors of LTS in COVID-19 patients can be both extrinsic and intrinsic: the time of intubation (the sequelae of prolonged intubation have long been recognized but are of particular concern in the COVID-19 pandemic due to aggressive cuff overinflation), the drugs used (as protectors, like corticosteroids), the use of prone position, comorbidities (diabetes, obesity). In any way, the common additional risk factor seems to be the inflammatory state of the upper airways linked to the viral infection, which also probably predisposes to an increased tendency to relapsing stenosis.

## Conclusion

In our COVID-19 related LTS series, we adopted a specific treatment for each patient, to offer the best treatment based on the state of their disease.

A later-intubation strategy requiring NIV to avoid invasive mechanical ventilation, and careful manipulation during PP, associated with better medical treatment by corticosteroids and other anti-inflammatory drugs may reduce the incidence of LTS. The early diagnosis and referral of patients to centers of reference for tracheal diseases seems essential for optimal management. Surgical resection remains the radical treatment of choice, provided that the inflammation is controlled, and that corticosteroid treatment is stopped to avoid complications related to the healing of the tracheal anastomosis. A conservative approach with medical and endoscopic treatment should be preferred in case of persistence of local airways inflammation.

Further studies with a larger sample of patients would allow us to better understand the disease, figure out how to prevent it and how to improve its treatment.

## Data Availability Statement

The original contributions presented in the study are included in the article/supplementary material, further inquiries can be directed to the corresponding author/s.

## Ethics Statement

Written informed consent was obtained from the individual(s) for the publication of any potentially identifiable images or data included in this article.

## Author Contributions

IO and EM had full access to all the data in the study and takes responsibility for the integrity of the data. NB, DR, OF, PG, MK, YU, and EZ contributed substantially to the study design, data analysis and interpretation, and the writing of the manuscript. All authors contributed to the article and approved the submitted version.

## Conflict of Interest

The authors declare that the research was conducted in the absence of any commercial or financial relationships that could be construed as a potential conflict of interest.

## Publisher's Note

All claims expressed in this article are solely those of the authors and do not necessarily represent those of their affiliated organizations, or those of the publisher, the editors and the reviewers. Any product that may be evaluated in this article, or claim that may be made by its manufacturer, is not guaranteed or endorsed by the publisher.
